# Experimental Researches upon Febrile Caloricity, Both before and after Death

**Published:** 1845-06

**Authors:** 


*Experimental Researches upon Febrile Caloricity, both before and after Death.—Post Mortem Fever.* By BENNET DOWLER, M. D., of New Orleans. (Western Journal of Medicine and Surgery, for June and October, 1844.)

This is an exceedingly interesting communication, which we have just received from the author, and to which we call the attention of the physiologist and the pathologist. It is the common opinion, that after the cessation of life, the body refrigerates according to the same laws as an inorganic substance of the same temperature, and placed in analogous circumstances. Yet the researches of Dr. Dowler show, that after death the heat may actually rise, and there may be what he terms "*post mortem* fever." We have only space to insert the following, which designates a "faint outline" of several cases, "selected from more than two hundred histories of temperature." They certainly authorise the deductions of Dr. Dowler, and lead to serious reflection.

"History 1st.—Highest temperature during life in the axilla. 104°; 10 minutes after death axilla 109°; in 15 minutes the thigh gives 113°, 9 above the living maximum; in 20 minutes the liver gives 112°; in 1 hour 40 minutes heart 109; thigh, old incision, 109°; in 3 hours after removing all the viscera, a new incision in the thigh gave 110°, 6° above the living maximum.

"Hist. 2d.—Alive: axilla 100°, hand 91°; dead 1 hour and 6 minutes: axilla, at the end of every 5 minutes, 102°, 104°, 107, thigh, 107°; 2 hours after death centre of the left lung 106°, apex 104°, heart 104°, thigh 106°; 3 hours, axilla 104°, liver

106°, thigh 106°—repeated, 106°, rectum 105°; 23 hours after death, room 90°, thigh 88°; putrefaction developed.

"Hist. 3d.—Last stage, hand 91°, axilla 100°; dead 30 minutes, axilla 104°; perineum, without incision, 102°; rectum 102°; epigastrium 103°; brain, through the orbit, 102°; body appeared to be growing hotter, when demanded by friends for interment.

Hist. 4th.—Last stage, hand 70°, (7° less than the air); axilla 95°; about 2 hours after death, axilla 100°, rectum 104°; axilla rose to 102°, epigastrium 101°, thigh 102°, brain 99°, heart and left chest 100°.

"Hist. 5th.—2 days before death, hand 101°, axilla 104°; 1 day before death, hand 100°, axilla 100°; dead 5 minutes, axilla gave at different periods, and was still rising, 103°-4°-5°-6°, epigastrium 106°, brain 101°, and falling; the thigh 101°, though exposed to a cold wind, which sunk the mercury 10° during the observations.

"Hist. 6th.—4½ hours after death, thigh exceeded the brain 6°, the chest 3°.

"Hist. 7th.—Air cold, (Oct. 26,) mercury falling 3 or 4° per hour; dead 1 hour, axilla 103°, epigastrium 104°, nearly, liver 108° and rising, brain 88°, falling; 2 hours after death, thigh 104°, left chest 97°."

From Dr. Dowler's experiments it seems, that the maximum of post mortem heat has been observed in the thigh. The following table contains five maxima of the eight principal regions in different subjects.

| Thigh. | Epigas'm. | Axilla. | Chest. | Heart. | Brain. | Rectum. | Liver. |
|---|---|---|---|---|---|---|---|
| 113° | 111° | 109° | 107° | 109° | 102° | 111° | 112° |
| 109° | 110° | 109° | 106.5° | 106° | 101° | 109° | 109° |
| 109° | 109° | 108° | 106° | 105° | 101° | 107° | 108° |
| 109° | 109° | 108° | 106° | 104° | 100° | 107° | 107° |
| 108° | 109° | 107° | 105° | 104° | 99° | 106° | 106° |
| M. 109.4° | 109.6° | 108.2° | 106.1° | 105.6° | 100.6° | 108° | 108.4° |

We have said that these experiments merit the attention of the physiologist; and we agree with Dr. Dowler that they are sufficient to overthrow the doctrine,—entertained, however, by but a few—

which ascribes the origin of animal heat solely to respiration. We do not doubt the accuracy of Dr. Dowler's results. They will, however, be repeated under competent auspices in this region. Fortunately we cannot test them on yellow fever subjects, nor have we many fatal cases—at present—of any form of fever. It will be interesting, however, to repeat them over an extensive field, and to determine whether the circumstances observed by Dr. Dowler may not exist universally. We shall, doubtless, have occasion to refer to this subject hereafter.

